# Intrauterine Exposure to Environmental Pollutants and Body Mass Index during the First 3 Years of Life

**DOI:** 10.1289/ehp.0800003

**Published:** 2008-10-08

**Authors:** Stijn L. Verhulst, Vera Nelen, Elly Den Hond, Gudrun Koppen, Caroline Beunckens, Carl Vael, Greet Schoeters, Kristine Desager

**Affiliations:** 1 Department of Pediatrics, University of Antwerp, Antwerp, Belgium;; 2 Provincial Institute for Hygiene, Antwerp, Belgium;; 3 VITO (Flemish Institute of Technological Research), Mol, Belgium;; 4 Department of Biomedical Sciences, University of Antwerp, Antwerp, Belgium;; 5 Center for Statistics, Hasselt University, Hasselt, Belgium;; 6 Department of Microbiology, University of Antwerp, Antwerp, Belgium

**Keywords:** body mass index, childhood, dichlorodiphenyldichloroethylene, dioxin-like compounds, hexachlorobenzene, obesity, polychlorinated biphenyls

## Abstract

**Objective:**

We investigated the association between body mass index (BMI) standard deviation score (SDS) and prenatal exposure to hexachlorobenzene, dichlorodiphenyldichloroethylene (DDE), dioxin-like compounds, and polychlorinated biphenyls (PCBs).

**Methods:**

In this prospective birth cohort study, we assessed a random sample of mother–infant pairs (*n* = 138) living in Flanders, Belgium, with follow-up until the children were 3 years of age. We measured body mass index as standard deviation scores (BMI SDS) of children 1–3 years of age as well as pollutants measured in cord blood.

**Results:**

DDE correlated with BMI SDS, with effect modification by maternal smoking and the child’s age. At 1 year, children of smoking mothers had higher BMI SDS than did children of nonsmoking mothers. At 3 years, this difference was reduced because of the faster rate of decline in BMI SDS in the former group. This relationship held except for children with high levels of DDE. DDE had a small effect on BMI SDS at 3 years of age in children of nonsmoking mothers (difference in BMI SDS for DDE concentrations between the 90th and 10th percentiles = 0.13). On the other hand, smoking enhanced the relation between DDE and BMI SDS at 3 years (difference in BMI SDS for DDE concentrations between the 90th and 10th percentiles = 0.76). Increasing concentrations of PCBs were associated with higher BMI SDS values at all ages (parameter estimate = 0.003 ± 0.001; *p* = 0.03).

**Conclusion:**

In this study we demonstrated that intrauterine exposure to DDE and PCBs is associated with BMI during early childhood. Future studies are warranted to confirm our findings and to assess possible mechanisms by which these pollutants could alter energy metabolism.

The prevalence of childhood obesity is reaching epidemic proportions worldwide (Kelishadi 2007). Furthermore, obese children and adolescents are at risk of becoming obese adults (Guo et al. 2002; Whitaker et al. 1997). This increased risk exists even for preschool-age children (Nader et al. 2006; Sachdev et al. 2005). Obese children and adolescents can develop serious comorbidities, including type 2 diabetes and metabolic syndrome (Sinha et al. 2002; Weiss et al. 2004). Although the main accepted cause of obesity is a genetic predisposition coupled with overeating and a lack of physical activity, there is still much uncertainty related to its etiology. For instance, it has been hypothesized that early environmental influences may contribute to the development of obesity. This hypothesis is illustrated by Toschke et al. (2002), whose study associated maternal smoking during pregnancy with childhood obesity. Therefore, it seems possible that chemical toxicants may also contribute to the etiology of childhood obesity. Recent reviews support the hypothesis that even brief exposures early in life to environmental endocrine-disrupting chemicals may increase one’s body weight (Newbold et al. 2007, 2008). A number of chemicals could indeed deregulate endocrine set points, influencing growth and development later in life. These chemicals include the pesticides dichlorodiphenyldichloroethylene (DDE) and hexachlorobenzene, dioxin-like compounds, and polychlorinated biphenyls (PCBs). DDE is the main metabolite of the pesticide DDT (dichlorodiphenyltrichloroethane). Although the use of DDT is prohibited in most countries, it is known to accumulate in the air, soil, and water, and it is biomagnified in the food chain [Agency for Toxic Substances and Disease Registry (ATSDR) 2007]. Until the early 1970s, hexachlorobenzene was used as a pesticide and in the production of fireworks, ammunition, and synthetic rubber. Currently, it is a by-product created by several processes. Furthermore, it breaks down very slowly and accumulates in animals, wheat, and vegetables (ATSDR 2007). PCBs and dioxins are halogenated hydrocarbons and highly persistent environmental pollutants. Dioxins are by-products from incineration processes and pesticide production, are not intentionally produced, and have no direct commercial value. There are no known natural sources of PCBs, which have been used as coolants and lubricants in electrical equipment. The industrial manufacturing of PCBs has been essentially prohibited since 1977, but, similar to dioxins, they are unintentionally formed as by-products from many chemical and thermal reactions (ATSDR 2007; Lundqvist et al. 2006). DDE, hexachlorobenzene, PCBs, and dioxins are all considered endocrine disruptors, defined as chemical substances that mimic or affect levels of endocrine mediators (Lundqvist et al. 2006). Indeed, studies have shown that these toxicants may interfere with thyroid hormone (Brucker-Davis 1998; Koppe et al. 2006; Takser et al. 2005), sex hormones (Jurewicz et al. 2006; Kelce and Wilson 1997; Koppe et al. 2006), and glucose metabolism (Lee et al. 2007; Lelli et al. 2007; Vasiliu et al. 2006).

To date, few prospective studies have investigated the association between prenatal exposure to chemicals and body mass index (BMI) (Gladen et al. 2000, 2004; Karmaus and Eneli 2004). In this study we investigated the association between BMI during the first 3 years of life and prenatal exposure to environmental pollutants hexachlorobenzene, DDE, dioxin-like compounds, and PCBs. We performed the analysis in three steps.

First, we analyzed the association between the pollutants and BMI during childhood. Because this was a longitudinal study, we also assessed the interaction with age. Furthermore, we assessed effect modification by sex because of possible sex-specific effects (Gladen et al. 2000) and possible effect modification by maternal smoking (Sagiv et al. 2007). Second, if a pollutant was associated with BMI, we then checked for a possible influence on birth weight or length to assess whether the influence of the pollutant on childhood growth already started from birth. Third, we checked whether the pollutant influenced height and/ or weight between 1 and 3 years of age.

## Materials and Methods

### Study design

In Flanders, the northern part of Belgium, we conducted a large-scale human biomonitoring study as part of the Flemish Centre of Expertise on Environment and Health, commissioned, financed, and steered by the Flemish government (Department of Science, Department of Public Health, and Department of Environment). A total of 1,196 mothers and their newborns were enrolled via 26 maternity wards in Flanders between September 2002 and February 2004. We selected these maternity wards through stratified sampling. We defined the “strata” as geographic areas in Flanders with different environmental and pollution characteristics: two urban areas (Antwerp and Ghent), an area characterized by fruit orchards, a rural area, and four industrial areas. In each of these areas, we selected an average of four maternity wards (one per season), from each of which we expected to recruit 50 participants. Present data are from a longitudinal substudy of a random sample of mothers living in the urban area of Antwerp or in rural Flanders.

Inclusion criteria included living for at least 5 years in the area of interest, being able to fill out questionnaires in Dutch, vaginal delivery at term, and an uncomplicated perinatal period (no neonatal or maternal distress, congenital malformations, or neonatal intensive care unit admission). During the week after delivery, field nurses visited the women in the maternity ward to check for participation eligibility, give information on the campaign, and collect completed questionnaires. These questionnaires collected information on the parents, including health status, smoking behavior, age, family composition, socioeconomic status, and the height and weight of the parents. We created two variables for smoking: smoked ever (smoked before or during pregnancy) and smoked during pregnancy. Next, we sent postal questionnaires every 6 months for children between the ages of 3 weeks and 36 months, collecting information about the child’s demography, diet, and health status and whether the child was breast-fed. We defined breast-feeding as being breast-fed for at least 1 day. We then created a separate covariate for children who had been breast-fed for at least 6 months. We used household income as an indicator of socioeconomic status: We defined low income as < € 1,240/month. The study protocol was approved by the medical ethics committees at the University of Antwerp. All parents gave written informed consent.

### Analysis of height and weight

We collected data on height and weight of the children by questionnaires administered at birth and at 12, 18, 24, 30, and 36 months of age. Parents filled in the height and weight measurements for their children obtained during physician visits or with Child and Family Services. We calculated BMI (kilograms per square meter) from questionnaire responses. We further analyzed BMI as BMI standard deviation scores [BMI SDS = (child’s BMI – population mean BMI)/population standard deviation], using Flemish growth curves (Hauspie and Roelants 2004) (Auxology 1.1; Pfizer, Brussels, Belgium). We used BMI SDS because BMI in children is highly dependent on the child’s age and sex and in order to linearize the repeated BMI measurements.

### Blood collection

We received at least 30 mL of cord blood from each participating birth mother. The cord blood was collected by the participating maternity ward personnel and than aliquoted. We separated plasma by centrifugation in the maternity laboratories within 24 hr of its collection, stored samples under refrigeration, and transported them in cool boxes to the analytical laboratory within 1 week of collection. We stored the plasma samples at the analytical laboratory at −20°C until analysis.

### Measurements in cord blood

Two laboratories measured PCBs (congeners 118, 138, 153, 170, and 180, because of their reported abundance in cord serum samples) and chlorinated pesticides by gas chromatography equipped with an electron capture detector using the method of Gomara et al. (2002). All analyses containing PCBs controlled for possible differences between both laboratories. Both laboratories participated in the Arctic Monitoring and Assessment Program (AMAP) proficiency testing scheme (Institut National de Santé Publique, Québec, Canada). Precision (relative standard deviation), estimated using results of the ClinChek and AMAP samples, ranged between 6.7% and 9.3% for all compounds, except for hexachlorobenzene (18.1%). The limit of detection for all chlorinated compounds in plasma was 0.02 μg/L. We assessed exposure to dioxin-like compounds via the dioxin-responsive chemical-activated luciferase expression (DR-CALUX) assay, based on the *in vitro* activation of the aryl hydrocarbon receptor in cultured H4IIE rat hepatoma cells by dioxin-like compounds extracted from 5 mL of cord plasma (BioDetection Systems BV, Amsterdam, the Netherlands). We performed extraction and cleanup procedures as described elsewhere (Koppen et al. 2001). The limit of detection was 0.14 ± 0.07 pg/mL extract or 14 pg CALUX-TEQ (toxic equivalents)/g fat for a plasma sample with a fat content of 200 mg/dL. We express all studied compounds on a lipid weight basis, because most of these pollutants are stored mainly in body fat and because we can reasonably assume that most of our blood samples result from fasting conditions. We simultaneously and gravimetrically determined the total plasma lipid concentration using the CALUX assay. If we obtained no value, we calculated the total lipid concentration based on routinely measured triglycerides and total cholesterol using the following formula: total lipids = 1.33 × (triglycerides + cholesterol) + 50.5 mg/dL (Covaci et al. 2006).

### Statistical analysis

We present all data as mean ± SD, median, and range, or as percentages. We used SAS version 9.1 (SAS Institute Inc., Cary, NC, USA) for all analyses. We calculated Spearman correlation coefficient between the pollutants and possible confounders. We balanced the initial design of the study with questionnaires being distributed every 6 months. However, the dates responding to the last measurements of height and weight were highly variable, resulting in an unbalanced design. Furthermore, nonresponses to distributed questionnaires complicated the analysis. Therefore, we used linear mixed models (a likelihood-based method), which provides valid results under less restrictive assumptions concerning missing data (missing at random) (Verbeke and Molenberghs 2001). The outcome variables included BMI SDS, weight SDS, and height SDS for children between 1 and 3 years of age. Mixed models determined which covariates significantly influenced the intercept and slope of, for instance, BMI SDS for this age range. We analyzed the outcome variables birth weight and birth length SDS using a multiple regression analysis. We also calculated least-square means.

We constructed single-pollutant models because of their high intercorrelation. We assessed the interaction of sex and smoking before and during pregnancy for each pollutant. We deleted nonsignificant interactions from the model. Initial analyses also contained the following covariates: age of the child, BMI of both parents, maternal age at time of birth, birth weight SDS, any instance of breast -feeding, maternal smoking before and/or during pregnancy, and household income. As covariates, models for weight and height SDS contained the weight and length of parents instead of their BMI. In every mixed model, we assessed the interaction of all covariates with time. We deleted nonsignificant interactions from the model. We applied a backward selection procedure (*p* = 0.05 as cutoff value), in view of the high number of variables and possible interactions.

We applied multiple imputation to investigate the stability of the results due to missing data. For multiple imputations, we replaced each missing value with a set of numbers (Markov chain Monte Carlo method) (Verbeke and Molenberghs 2001). We analyzed the results of each imputation as complete data sets and combined the results of these analyses. We defined statistical significance as *p* < 0.05.

## Results

### Subject characteristics

Table 1 presents subject characteristics (*n* = 138). The percentages of missing data for BMI SDS at 12, 18, 24, 30, and 36 months were 5, 4, 24, 38, and 40%, respectively, and the estimated mean BMI SDS values ± SE were 1.08 ± 0.87, 0.87 ± 0.89, 0.20 ± 0.98, 0.02 ± 0.89, and −0.32 ± 0.79, respectively. Table 2 presents results of a Spearman correlation analysis between the pollutants and possible confounders. All pollutants are significantly intercorrelated: Correlation coefficients among DDE, PCBs, and hexachlorobenzene are between 0.6 and 0.7. The correlation between the latter pollutants and dioxin-like compounds are between 0.2 and 0.3.

### Association of chemical toxins with BMI SDS for children between 1 and 3 years

Table 3 presents the model for the association between DDE and BMI SDS. The parameter estimates and *p*-values of this model did not change when we excluded birth weight SDS from this analysis. The model is complex because of significant interactions among DDE, smoking ever, and child’s age. Table 4 further outlines the results of the model with least-square means of BMI SDS calculated according to the mother’s smoking status, for various ages of the child (1, 2, and 3 years) and levels of DDE (10th percentile, median, and 90th percentile). We can summarize the results for DDE as follows: At 1 year of age, children of smoking mothers had a higher BMI SDS compared with children of nonsmoking mothers. However, there was a nonsignificant trend such that this difference diminished as DDE levels increased. At the end of the study, at 3 years of age, the observed difference in children’s BMI SDS between smoking and non-smoking mothers was reduced, because of the faster rate of decline in BMI SDS in subjects of the former group. This relationship held for all children except those with high levels of DDE.

Finally, the effect of increasing DDE levels on BMI SDS at 3 years of age was small in children of nonsmoking mothers (the difference in BMI SDS for a DDE level of 450 ng/g and a DDE level of 63.7 ng/g = 0.13). On the other hand, smoking enhanced the effect of DDE on a child’s BMI SDS at 3 years of age (difference = 0.76).

In the multiple imputation analysis, the borderline significant interaction between age and DDE became insignificant (*p* = 0.3). The interaction among age, DDE, and maternal smoking ever remained significant. Furthermore, the effect of DDE on BMI SDS at 1 year of age increased (0.001 ± 0.001; *p* = 0.08).

Increasing concentrations of PCBs were associated with higher BMI SDS values between 1 and 3 years of age (Table 5). For PCBs, we found no significant interactions with sex and the smoking status of the mother. Results for PCBs did not change during the multiple imputation analysis. We also performed a similar analysis for the TEQ factor of the single dioxin-like PCB that we measured, PCB-118 (Van den Berg et al. 2006), but this factor was not correlated with BMI *Z*-score. Finally, we found no association between BMI SDS and hexachlorobenzene and dioxin-like compounds.

### Associations at and after birth between DDE and PCBs levels and weight and length SDS

Both maternal smoking and PCBs were associated with birth weight SDS, although their effects depended on each other (Tables 6 and 7). We observed similar effects for birth length SDS (Tables 6 and 7). DDE was not associated with birth weight or length.

In a model similar to the analysis presented in Table 3, including weight SDS for children between 1 and 3 years as an outcome variable, DDE was not associated with weight SDS at 1 year of age (*p* = 0.5). We found indications that smoking ever was associated with increased weight SDS at 1 year of age (β ± SE = 0.653 ± 0.348; *p* = 0.06). Similar to data presented in Table 3, we found significant interactions between maternal smoking and age (−0.253 ± 0.129; *p* = 0.045), DDE and age (0.001 ± 0.000; *p* = 0.05), and maternal smoking and DDE and age (0.001 ± 0.000; *p* = 0.04). PCBs did not influence weight SDS.

Finally, PCBs were associated with a systematic lower length SDS for children between 1 and 3 years of age (−0.002 ± 0.001; *p* = 0.04). DDE was not associated with length SDS.

## Discussion

In this study we found that DDE had a small association with BMI SDS in 3-year-old children of nonsmoking mothers. However, this association was enhanced in children of smoking mothers. This relation seemed to be mediated by an influence on weight gain during the preschool period. Second, PCBs were also associated with higher BMI SDS values. This association was not modified by smoking of the mother, nor did it change with the age of the child. The association between PCBs and BMI SDS seemed to be mediated by a negative influence on birth weight and length and on length between 1 and 3 years.

Our results should be considered in light of some important study limitations. We found significant associations and interactions with maternal smoking ever, which includes mothers who smoked before pregnancy or during pregnancy, but not with smoking during pregnancy alone. This result could be attributable to insufficient power. Second, although it has been suggested that smoking could increase susceptibility to pollutants by interfering with immune function and by promoting oxidative stress, which could sensitize cells to pollutants, it is also known that mothers who quit smoking before pregnancy gain more weight during pregnancy (Favaretto et al. 2007), which could modulate both birth weight/length and cord blood pollutant levels. This could suggest that pharmacokinetic factors are responsible for these associations. Unfortunately, we did not record maternal weight gain during pregnancy in our study, which should be considered as a major study limitation. This could also suggest that maternal smoking has long-term health effects. These limitations require elucidation in future studies.

A second limitation of this study is that we related reported results to BMI SDS until a child reached 3 years of age. Although there is a correlation between BMI during pre-school age and adult BMI (Nader et al. 2006; Sachdev et al. 2005), we do not know whether the correlation with BMI will persist into later periods and whether these children will actually become overweight or obese. Third, although we found no differences in confounders between this cohort and the larger cohort from which it originates, there are significant regional differences in exposure to these pollutants in Flanders (Koppen G, Den Hond E, Nelen V, Van De Mieroop E, Bruckers L, Bilau M, et al., unpublished data). Therefore, our findings cannot be generalized to the entire Flemish population. Another limitation is that the exact duration of breast-feeding was not available, so we had to create two crude variables to assess postnatal exposure. Finally, as in every longitudinal study, missing data are a hindrance. However, we were able to confirm our results by multiple imputation.

The preschool period is a critical time in the development of obesity, because it coincides with the so-called adiposity rebound phenomenon. Indeed, BMI rises in infancy but declines during the preschool years, increasing again during the primary school years (Rolland-Cachera et al. 1984). Even though we did not assess adiposity rebound in this study, it is accepted that higher BMI SDS during this period is associated with an increased risk of obesity in adult life (Whitaker et al. 1998). Furthermore, there is a known correlation between BMI during the preschool years and adult BMI (Nader et al. 2006; Sachdev et al. 2005). This is the first study demonstrating that environmental pollution may influence BMI during these critical years. DDE and PCBs may influence BMI by interfering with several endocrine mediators. For instance, PCB and DDE levels are related to lower thyroid hormone levels in pregnant women (Takser et al. 2005). DDT may also have estrogenic, antiandrogenic, and antiprogestin effects (Jurewicz et al. 2006; Kelce and Wilson 1997). PCBs may exert estrogenic, antiandrogenic, or antiestrogenic effects (Koppe et al. 2006). Other studies provide evidence that PCBs are associated with increased insulin resistance in adults (Lee et al. 2007; Vasiliu et al. 2006). Although endocrine disruption caused by these pollutants has been well documented, few studies have investigated the influence of pre-natal exposure to pollutants and BMI later in life. One follow-up study found that increased transplacental exposure to DDE is associated with a significantly higher weight adjusted for height in adolescent boys (Gladen et al. 2000). However, a study of the same group in adolescent boys with higher exposure to DDT and DDE failed to replicate this finding (Gladen et al. 2004). A study by Karmaus and Eneli (2004) demonstrates that intrauterine exposure to DDE, but not to PCBs, is associated with a higher weight and BMI in adult females.

The association between PCBs and BMI in our cohort seemed to begin early, demonstrated by its association with birth weight and length. Furthermore, maternal smoking modified this effect. This finding is in agreement with a recent study conducted by Sagiv et al. (2007), who also found effect modification by smoking on the association between PCBs and birth weight. After 1 year of age, PCBs were associated with smaller heights. Hertz-Picciotto et al. (2005) found that prenatal PCBs were associated with increased growth in 5-year-old girls but not in boys. Lamb et al. (2006) found that the association between PCBs and childhood growth among boys depended on the degree of *ortho* substitution.

On the other hand, estimated effects of DDE appeared to manifest later in life, because we found no association with birth size. We did find an association with weight that depended on the child’s age and whether the mother smoked. It is important to emphasize that there was a trend for DDE effect modification by sex (data not shown), but the present study did not have sufficient power to render this effect significant. Another prospective study found that increased prenatal DDE concentrations were associated with decreased height at 1, 4, and 7 years of age (Ribas-Fito et al. 2006). Karmaus et al. (2002) demonstrated that growth during childhood was significantly reduced in girls with high DDE concentrations measured at 8 years of age. They observed no growth effect of DDE in boys. Contradictory results may be attributable to different degrees of exposure, the inclusion of premature children, the age of assessment of anthropometric measurements (prepubertal or pubertal children), and the number of assessed confounding variables.

Finally, familial influences have been confirmed as risk factors increasing BMI in children. The finding that increased birth weight is associated with higher BMI in early childhood has been previously documented (Li et al. 2007; Whitaker and Dietz 1998), as has the finding that exposure to prenatal smoking increases incidence of overweight children (Oken et al. 2008).

In conclusion, this study documents that intrauterine exposure to DDE and PCBs is associated with increased BMI during early childhood. The association with PCBs starts from birth, with a negative effect on birth weight and birth length, whereas the association with DDE is measurable between 1 and 3 years of age, with no effect on birth weight or length. Future prospective studies are needed to confirm these findings, including studies assessing possible mechanisms by which these pollutants could alter energy metabolism. Finally, we recommend more studies investigating the mechanisms of effect modification by smoking, especially on the influence of maternal weight gain during pregnancy.

## Figures and Tables

**Table 1:** Subject characteristics.

Variable	Value
No.	138
BMI at 1 year of age (kg/m^2^ )	17.2 ± 1.5
BMI SDS at 1 year of age (kg/m^2^ )	1.14 ± 1.23
BMI at 3 years of age (kg/m^2^ )	15.7 ± 1.8
BMI SDS at 3 years of age (kg/m^2^ )	−0.36 ± 1.56
Percent boys (%)	53.3
Percent from rural agglomerations (%)	38.7
Birth weight (kg)	3.4 ± 0.4
Birth weight SDS	−0.25 ± 0.92
Birth length (cm)	50.45 ± 1.78
Birth length SDS	0.07 ± 0.96
Age of mother (years)	30.8 ± 4.1
BMI of mother (kg/m^2^ )	23.19 ± 3.95
BMI of father (kg/m^2^ )	24.25 ± 3.12
Households with low income (%)	13.6
Maternal smoking ever (%)	44.4
Maternal smoking during pregnancy (%)	9.6
Breast-feeding ever (%)	66.4
Breast-feeding for at least 6 months (%)	23.4
Hexachlorobenzene (ng/g lipids)	34.4 ± 19.0 (range, 4.3–108.3)
DDE (ng/g lipids)	212 ± 243 (range, 24–1,816)
Dioxin-like compounds (pg CALUX-TEQ/g lipids)	31.0 ± 19.2 (range, 6.0–78.7)
PCBs (ng/g lipids)	117 ± 76 (range, 9–442)

Values are mean ± SD or percent.

**Table 2:** Spearman correlation analysis between pollutants and possible confounders.

Possible confounder	DDE	Hexachlorobenzene	PCBs	Dioxin-like compounds
Female sex	−0.04	0.00	−0.01	−0.02
Rural Flanders	0.16	0.19*	−0.01	−0.08
Age of mother (years)	0.29**	0.23*	0.40**	−0.07
Birth weight SDS	−0.00	−0.04	−0.13	−0.03
BMI of mother (kg/m^2^ )	0.03	0.10	−0.22*	−0.10
BMI of father (kg/m^2^ )	−0.02	0.08	−0.01	0.13
Low socioeconomic status	−0.10	−0.05	0.17	0.17
Maternal smoking ever (%)	−0.17	−0.17	0.03	−0.11
Maternal smoking during pregnancy (%)	0.00	0.08	0.11	−0.09

* *p* < 0.05;

** *p* < 0.01.

**Table 3:** The association between DDE and BMI SDS of children between 1 and 3 years of age.

Parameter	β ± SE	*p*-Value
Intercept	2.643 ± 0.298	< 0.001
Birth weight SDS	0.310 ± 0.095	0.001
Smoking ever	1.259 ± 0.378	0.001
DDE (ng/g lipids)	−0.002 ± 0.001	0.2
DDE × maternal smoking ever	−0.003 ± 0.001	0.06
Age of the child	−1.116 ± 0.146	< 0.001
Maternal smoking ever × age of the child	−0.497 ± 0.188	0.01
DDE × age of the child	0.001 ± 0.001	0.047
Maternal smoking ever × DDE × age of the child	0.001 ± 0.001	0.04

**Table 4:** Least-square means for BMI SDS computed according to maternal smoking status, for different levels of DDE (10th and 90th percentiles and median) at the ages of 1, 2, and 3 years.

	63.7 ng/g lipid (10th)		138.9 ng/g lipid (median)		450.0 ng/g lipid (90th)	
Child’s age	Smoking	Mean ± SE	Smoking	Mean ± SE	Smoking	Mean ± SE
1 year	No	0.74 ± 0.15	No	0.80 ± 0.13	No	1.03 ± 0.15
	Yes	1.42 ± 0.17	Yes	1.38 ± 0.14	Yes	1.21 ± 0.30
2 years	No	0.11 ± 0.14	No	0.15 ± 0.13	No	0.32 ± 0.15
	Yes	0.38 ± 0.17	Yes	0.43 ± 0.14	Yes	0.65 ± 0.30
3 years	No	−0.53 ± 0.21	No	−0.50 ± 0.19	No	−0.39 ± 0.20
	Yes	−0.66 ± 0.24	Yes	−0.51 ± 0.20	Yes	0.10 ± 0.40

**Table 5:** The association between PCBs and BMI SDS between 1 and 3 years.

Parameter	β ± SE	*p*-Value
Intercept	0.758 ± 0.588	0.2
Birth weight SDS	0.333 ± 0.045	0.001
Maternal smoking ever	0.368 ± 0.172	0.03
Maternal BMI (kg/m^2^ )	0.046 ± 0.022	0.04
PCBs (ng/g lipid)	0.003 ± 0.001	0.03
Age of the child	−0.766 ± 0.069	< 0.001

**Table 6:** Association between PCBs and birth weight and birth length SDS.

Parameter	β ± SE	*p*-Value
Birth weight SDS		
Intercept	−1.088 ± 0.537	0.05
Maternal smoking ever	0.369 ± 0.152	0.02
Paternal weight (kg)	0.014 ± 0.006	0.03
PCBs (ng/g lipids)	−0.002 ± 0.001	0.03
PCBs × maternal smoking ever	−0.003 ± 0.001	0.004
Birth length SDS		
Intercept	−12.244 ± 2.780	< 0.001
Maternal smoking ever	0.327 ± 0.156	0.04
Paternal height (m)	0.037 ± 0.012	0.002
Maternal height (m)	0.036 ± 0.013	0.006
PCBs (ng/g lipid)	−0.002 ± 0.001	0.08
PCBs × maternal smoking ever	−0.003 ± 0.001	0.02

**Table 7:** Least-square means for birth weight and birth length SDS computed according to maternal smoking status and for different levels of PCBs (10th and 90th percentiles and median).

	48.6 ng/g lipid (10th)		95.5 ng/g lipid (median)		197 ng/g lipid (90th)	
Measure	Smoking	Mean ± SE	Smoking	Mean ± SE	Smoking	Mean ± SE
Birth weight SDS	No	−0.29 ± 0.14	No	−0.25 ± 0.11	No	−0.17 ± 0.15
	Yes	0.13 ± 0.17	Yes	−0.13 ± 0.13	Yes	−0.69 ± 0.18
Birth length SDS	No	0.04 ± 0.14	No	0.08 ± 0.11	No	0.15 ± 0.16
	Yes	0.41 ± 0.17	Yes	0.19 ± 0.13	Yes	−0.27 ± 0.18
